# The community and consumer food environment and children’s diet: a systematic review

**DOI:** 10.1186/1471-2458-14-522

**Published:** 2014-05-29

**Authors:** Rachel Engler-Stringer, Ha Le, Angela Gerrard, Nazeem Muhajarine

**Affiliations:** 1 Department of Community Health and Epidemiology, College of Medicine, University of Saskatchewan, 107 Wiggins Road, Saskatoon, Saskatchewan, Canada; 2 University Library, University of Saskatchewan, 3 Campus Drive, Saskatoon, Saskatchewan, Canada

## Abstract

**Background:**

While there is a growing body of research on food environments for children, there has not been a published comprehensive review to date evaluating food environments outside the home and school and their relationship with diet in children. The purpose of this paper is to review evidence on the influence of the community and consumer nutrition environments on the diet of children under the age of 18 years.

**Methods:**

Our search strategy included a combination of both subject heading searching as well as natural language, free-text searching. We searched nine databases (MEDLINE, Web of Science, CINAHL, Embase, Scopus, ProQuest Public Health, PsycINFO, Sociological Abstracts, and GEOBASE) for papers published between 1995 and July 2013. Study designs were included if they were empirically-based, published scholarly research articles, were focused on children as the population of interest, fit within the previously mentioned date range, included at least one diet outcome, and exposures within the community nutrition environment (e.g., location and accessibility of food outlets), and consumer nutrition environment (e.g., price, promotion, and placement of food choices).

**Results:**

After applying exclusion and inclusion criteria, a total of 26 articles were included in our review. The vast majority of the studies were cross-sectional in design, except for two articles reporting on longitudinal studies. The food environment exposure(s) included aspects of the community nutrition environments, except for three that focused on the consumer nutrition environment. The community nutrition environment characterization most often used Geographic Information Systems to geolocate participants’ homes (and/or schools) and then one or more types of food outlets in relation to these. The children included were all of school age. Twenty-two out of 26 studies showed at least one positive association between the food environment exposure and diet outcome. Four studies reported only null associations.

**Conclusions:**

This review found moderate evidence of the relationship between the community and consumer nutrition environments and dietary intake in children up to 18 years of age. There is wide variation in measures used to characterize both the community and consumer nutrition environments and diet, and future research should work to decrease this heterogeneity.

## Background

We know that food choice is not simply an individual behaviour, but a practice that is influenced by the social and physical environments [1], and that these environments vary greatly, contributing to growing nutritional health inequalities. North American environments generally promote food that is packed with calories (energy-dense food), and offer little incentive for living an active lifestyle [2], particularly in low income neighbourhoods (NBH) [3]. According to findings from the Canadian Health Measures Survey (CHMS), childhood obesity, which has increased significantly since 1981 due to rising levels of body fat [4], has been associated with various health problems that continue throughout the person’s lifespan.

The food environment, broadly conceptualized to include any opportunity to obtain food [5], is increasingly being recognized as critical to health [6-9]. The food environment is multidimensional [7,10] and according to Glanz et al. [7], includes four aspects: (1) community nutrition environment (e.g., location and accessibility of food outlets), (2) consumer nutrition environment (e.g., price, promotion, and placement of food choices), (3) organizational nutrition environment (access to food in other settings such as workplaces and schools), and (4) information environment (marketing, media, advertising). In this article we will use the terms community and consumer nutrition environment interchangeably with community and consumer food environment.

Research on food environments is currently growing quickly. For example, in 2011 and 2012 three systematic reviews of literature on the food environment (FE) were published, one examining the relationship between aspects of the FE and diet across the population [11], another between aspects of the FE and overweight and obesity in children [12], and a third that reviewed studies of fast food access in various populations [13]. A recent systematic review examines the dietary assessment methods used in food environment studies [14].

Glanz et al. [7] argued in 2005 that more research was needed specifically on the community and consumer nutrition environments for two major reasons. First, they were the most under-studied, and second, they are likely to have the largest impact on nutritional health. The literature on community and consumer nutrition environments has grown dramatically since then as reflected in the systematic reviews mentioned above, but there continue to be major gaps. First, the majority of the literature to date has not focused on dietary outcomes, but rather on body weight (using BMI), a more distal outcome that is a reflection of much more than diet. This makes it difficult to separate any specific impacts on diet from those of physical activity, for example. Second, the literature to date has typically been focused on adults, yet we know that food and eating practices are established in childhood [15], and that children navigate and understand their food environments in their own ways [16,17] and it is therefore important to better understand the impacts of the food environment on diet in children specifically so that interventions can be tailored to prevention in this population group.

Osei-Assibey et al. [12] examined an important aspect of the FE-health relationship in children, that is FE and overweight/obesity, but this study did not cover the FE impact on nutrition behaviour specifically. Therefore, in order to better understand the link between food environment and nutrition, this systematic review addresses the following specific question: How do community and consumer food environments (as defined by Glanz et al. [7]) influence children’s diet?

## Methods

Public health research often extends beyond the traditional health or biomedical fields and is frequently indexed in other disciplines such as the social sciences (including economics, sociology, human geography, etc.). Specifically, the interdisciplinary nature of community and consumer nutrition environments dictated the need to search beyond the scope of conventional medical databases. An initial lengthy list of databases was suggested and reviewed by members of the research team. These databases were grouped into three tiers by their relevance and the tier one databases identified were chosen as the most appropriate to search for the systematic review. These nine databases included MEDLINE, Web of Science, CINAHL, Embase, Scopus, ProQuest Public Health, PsycINFO, Sociological Abstracts, and GEOBASE.

The database searching was undertaken between July 24 and 26, 2013. Overall the search strategy included a combination of both subject heading searching (dependent on the controlled vocabulary for each database) as well as natural language, free-text searching. The original search strategy was developed in MEDLINE (see Additional file 1) and was reviewed and refined by the research team. The search strategy was then adapted to the other databases (see Additional file 2 for comprehensive listing of search strategies). The search strategy was quite lengthy as dictated by the complexity of the research topic. The main search concepts were based on the community OR consumer food environments AND children AND diet. The community food environment focuses on the classification of food outlets (specifically number, type, location, and accessibility of food sources) while the consumer food environment observes the characteristics within and around these food sources including portion sizes, food options, placement, price, and promotion of food. The search strategy focused on the specific population of children, defined as aged <18 years. The final search category, diet, proved to be complex due to multifaceted dietary factors ranging from eating (example: eating behavior), to specific dietary categories (such as sugar-sweetened beverages, dietary carbohydrates, etc.).

**Figure 1:**
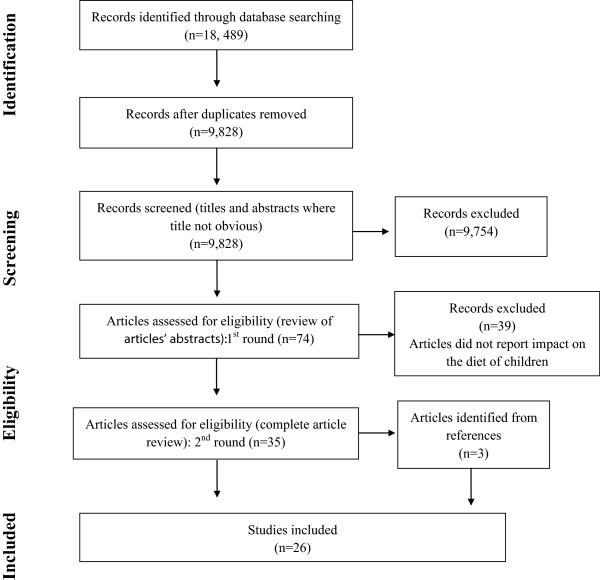
Screening process.

References were initially exported and sorted for each database search and were then merged into one EndNote library. The duplicate references were then systematically removed. The total number of references remaining after de-duplication was 9,848. These references then underwent a title and brief abstract review (in the case of articles that did not have obviously illustrative titles), based on a set of inclusion and exclusion criteria. Due to the time involved in reviewing such a large number of references, we were unable to have two people review each one. Instead, we divided the references into four groups and had two of the authors (RE-S and HL) plus two public health nutrition trained research assistants each review a quarter of the references. Reviewers were asked to be overly inclusive at this stage and to only remove references that were very clearly not relevant based upon their title and a cursory abstract review. There were 74 references remaining after the first round of review. A second review was undertaken where all abstracts were reviewed and 35 research articles remained. These articles were reviewed in their entirety and a few additional exclusions were made and 23 articles remained. The final stage of the literature search involved hand-searching the bibliographies of the remaining articles which uncovered an additional 3 articles for inclusion for a final total of 26 articles. Figure 1 represents the screening process.

The systematic literature search resulted in large set of references to review. While this is not ideal, it is also not uncommon considering the interdisciplinary nature of the research question (initial large reference sets have also been demonstrated in some of the systematic reviews mentioned above). The clear inclusion and exclusion criteria enabled the research team to efficiently review the references. The inclusion criteria were as follows: i) children under 18 years of age were identified as the population of interest; ii) focus on food environments outside the home but not including the organizational nutrition environments; iii) focus on some aspect of diet; iv) empirically-based, scholarly research articles (not review articles); v) English language; vi) date range of 1995 to present. That year was chosen because prior to then there is virtually no published literature in this area. References were excluded where the subject matter discussed food environments other than the community or consumer nutrition environments as defined by Glanz et al. [7]. These included the organizational nutrition environment (home, schools, daycare centres) and the information environment (example: television advertising). There were a large number of references that focused on the school food environment. Other references were excluded because the populations studied were outside the scope of the review including the feeding of infants (breastfeeding, formula feeding) and the diet of pregnant women. Others were excluded because the research focused on overweight or obesity and not diet specifically. While many articles appeared to be on-target, a closer examination revealed the articles’ lack of focus on the impact on the diet of children.

## Results

In total, 26 articles met the inclusion criteria and were included in this review (Table 1). It is important to note that while our interest for this review is in examining the relationship between FE and diet, some of the articles we have included report other parameters, in addition to diet (BMI, socioeconomic characteristics, etc.). When other associations were included, we did not report on these as our focus remained in understanding the relationship between FE and diet related outcomes.

**Table 1:** Studies examining the relationship between community and consumer nutrition environment and diet in children

**Author (Year)**	**FE exposure**	**Diet outcomes (method employed)**	**Location**	**Age group**
An et al. [18]	Food outlet (FF restaurants, convenience stores, small food stores, grocery stores and large supermarkets) distribution at several distances (varying from 0.1, 0.5, 1,0 to 1.5 miles) from child’s home and school	Daily servings of FV, 100% juice, milk, soda, high-sugar foods and FF (self- reported)	USA (CA)	5-17 years
Buck et al. [19]	Food supply around school calculated using number of stores and restaurants divided by number of residents per area	Junk food and simple sugar food consumption per week (FFQ and 24 hour dietary recall)	Germany (Lower Saxony)	6-9 years
Davis & Carpenter [20]	Proximity and density of FF to school (a half-mile radius)	Soda, FV, juice, fried potato foods consumption in past 24 hours (self- reported)	USA (CA)	Middle school and high school students (no specific age reported)
Ding et al. [21]	Self-report proximity of food outlets to home	Daily FV intake per day (self-reported)	USA (San Diego, Boston, Cincinnati)	Two samples 5–11 and 12–18 years
Edmonds et al. [22]	FV, 100% juice availability and shelf space in food stores and restaurants around home	FV (including fried potatoes), juice consumption per day (24 hour recall)	USA (TX)	11-14 years (boys only)
Fraser et al. [23]	FF accessibility using 1000m buffer from home	FF (restaurant) consumption (self-reported)	UK (former Avon county)	13 years
He et al. [24]	Junk food density within 1km of home and school; distance from home and school to closest FF restaurant and convenience store	Overall diet quality – Healthy Eating Index (FFQ)	Canada (ON)	11-14 years
He et al. [25]	Junk food density within 1km of home and school; distance from home and school to closest FF restaurant and convenience store	Food purchasing behaviour (FF and convenience store) (self-reported)	Canada (ON)	11-14 years
Ho et al. [26]	Self-report presence of food outlets near home within 5 minute walking	Consumption of 4 food groups: high fat foods, junk food/soft drinks, fruit, and vegetables (FFQ)	Hong Kong	14.5 years (mean)
Jago et al. [27]	Distance to food outlets from home and density of food outlets within a 1-mile radius of the participant's home address	FV, 100% juice consumption (FFQ)	USA (TX)	10-14 (boys only)
Jennings et al. [28]	Food outlet (BMI-healthy and unhealthy) availability within 800m of home	Food group intake (Food and drink diary)	UK (Norfolk)	9-10 years
Khan et al. [29] *longitudinal	FF prices from the Cost of Living Index and FF outlet density	FF consumption in the past 7 days (self-reported)	USA (nationally representative sample)	5th graders in 2004 and 8th graders in 2007
Laminchhane et al. [30]	Accessibility and availability of supermarkets (4 & 6 miles) and FF (1 mile) to home	The overall dietary intake quality composed of 8 food groups (grains, vegetables, fruit, dairy, meat, nuts/seeds/legumes, fats/oils, sweets) and food group consumption (DASH Index)	USA (SC)	10-20 years (newly diagnosed with diabetes)
Laska et al. [31]	Distance and density of all food outlets to home and school (800, 1600 and 3000 m buffer zones)	Food group consumption (24 hour recall)	USA (MN)	10.8-17.7 years
Leung et al. [32]	“Food and retail” scale - Food outlet audit on random street segments within 0.25 mile of home	Total energy intake (24 hour recall)	USA (CA)	6.5-8.1 years
Longacre et al. [33]	Availability of FF outlets – onsite audit	FF intake in the past week (self-reported)	USA (NH and VT)	12-18 years
Mushi-Brunt et al. [34]	Grocery store availability and accessibility – GIS within census tract	FV intake (FFQ)	USA (Midwestern United States)	6-11 years
Pabayo et al. [35]	Food outlet availability within 1km of home	SSB intake (soft drink and fruit juice) (self-reported and Children Eating Behavior Questionnaire)	Canada (AB)	4-5 years
Powell et al. [36]	FV and FF prices from Cost of Living Index	Daily FV consumption (self-reported)	USA (nationally representative sample)	14.7 years (mean)
Skidmore et al. [37]	Distance to nearest food outlet, density/km^2^ of food outlets within 800 m buffer zone of a child’s home	Food choices (consisted of 15 common foods) (Health Behaviour in School Children (HBSC) questionnaire)	UK (Norfolk)	9-10 years
Smith et al. [38] *longitudinal	Density of food outlets within 400 and 800m network distance from school	Healthy vs unhealthy diet scores (self-reported)	UK (Newham, Hackney and Tower Hamlets)	12.2 years (mean)
Sturm & Datar [39]	Price indices for meat, FV, dairy and FF calculated from Cost of Living Index	Consumption of FV, milk, soft drinks and FF in the past 7 days (food consumption questionnaire)	USA (nationally representative sample)	11.2 years (mean)
Timperio et al. [40]	Availability of 5 types of food outlets within 800m buffer zone of home	Consumption of FV (self-reported)	Australia (Greater Melbourne and Geelong areas)	5-6 and 10–12 years
Timperio et al. [41]	Distance, density and availability of FF/takeaway within 800m of home and school	Consumption of FF/takeaway (self-reported)	Australia (Greater Melbourne and Geelong areas)	5-6 and 10–12 years
Veugelers et al. [42]	Access to food stores from home (poor to excellent)	Diet quality index, daily servings of FV, energy from fat (Harvard FFQ)	Canada (Nova Scotia)	10-11 years
Wang & Shi [43]	Self-reported NBH density of food outlets within 5 km	Macronutrient and calorie intake (food consumption questionnaire)	China (Guangxi, Guizhou, Heilongjiang, Henan, Hubei, Hunan, Jiangsu, Liaoning, and Shandong)	6-18 years

Dietary Approaches to Stop Hypertension: DASH.

Food Frequency Questionnaire: FFQ.

Fruit and Vegetables: FV.

Fast Food: FF.

Sugar-Sweetened Beverage: SSB.

Almost all of the articles (22) were published in the last five years, which is consistent with the general increasing interest in this research area. The vast majority of the studies were cross-sectional in design, except for two articles reporting on longitudinal studies [29,38]. The FE exposure(s) in most studies included aspects of the community FE, except for three that focused on the consumer FE [22,29,36]. The community FE characterization most often used Geographic Information Systems (GIS) to geocode study participants’ homes (and/or schools) and then one or more types of food outlets in relation to these. Half the studies (13) were conducted in the USA, 4 conducted in Canada, 5 studies conducted in Europe (4 from UK, 1 from Germany), 2 from Australia, 2 from Asia (one from China and one from Hong Kong). The children included in these studies were all school-aged children. Some were focused on a narrow age range, while others included children ranging in age from 5–18. The basic aspects of each article are reported in Table 1 above.

This table also highlights the wide range of assessment techniques that were used in the studies to calculate an array of dietary outcomes, including fruit and vegetable (FV) intake, fast food (FF) consumption, sugar-sweetened beverage (SSB) intake, dietary quality indices, as well as consumption of other food groups and macronutrients and energy. The most common dietary assessment technique was the self-reported method (n = 11 studies) to assess consumption patterns of specific foods, such as fruit and vegetable, milk or FF intakes. These studies used simple questions to collect information on food intake such as “In the past 7 days how many times did you eat FF, for example, food from McDonalds, Burger King, KFC, or Dunkin’s Donuts?” [33] or “How often do you eat at least some green vegetables?” and “How often do you eat at least some fruit?” [36]. Food Frequency Questionnaires (FFQ) were used in 6 studies. In general FFQs were the methods of assessment of choice for children aged 9 years and above, because at that age they are capable of completing them in a self-administered form [44]. Other methods of dietary assessment used were the 24 hour recall (4 studies), a food consumption questionnaire (FCQ) (2 studies), the Health Behavior in School Children questionnaire (1 study), Children’s Eating Behavior Questionnaire (1 study), a food and drink diary (1 study) and the Dietary Approaches to Stop Hypertension (DASH) index (1 study).

In Table 2 the 26 studies are listed by the approach they took for assessing the FE exposure. The most common technique used for measuring exposure was GIS based measures (16 studies). These GIS based measures captured the geographic relationship between residents’ home and/or school and healthy food outlets (grocery stores) – 1 study [34]; and unhealthy food outlets (FF and convenience stores) – 6 studies; and various food outlet types, including supermarkets, convenience stores, FF outlets – 9 studies. Four studies used participant reported measures to assess some dimension of food access, including perceived access to shops [42], perceived proximity to each type of food outlet [21], and some dimension of food availability, such as perceived availability of FF outlets [26] and perceived density of food outlets [43]. These measures were all single-item indicators. Three studies used pricing measures to examine the association between food price and food consumption such as FV, snack items, and FF [29,36,39]. The food price indices of these three studies were all computed from the American Chamber of Commerce Researchers Association Cost of Living Index reports. Two additional studies used a store audit measure [32,33]. The study by Longacre et al. [33] used an audit of the number of in-town FF outlets to assess the relationship between in-town FF outlets and FF intake. The other study, by Leung et al. [32], used street audit data to form a NBH “food and retail” scale to investigate if this was associated with total energy intake. Finally, one study used an inventory checklist of fruit, juice and vegetable availability [22].

**Table 2:** Studies cross-classified by method of food environment exposure measurement and type of diet outcome

**FE exposure diet outcome**	**GIS**			**Pricing**	**Self-report outlets**	**Food outlet audit**	**Fruit and vegetable availability in food outlets**
	**Healthy outlets**	**Unhealthy outlets**	**Various outlets**				
**Fruit and vegetable intake**	Mushi-Brunt et al. [34]		Jago et al. [27] Timperio et al. [40]	Powell et al. [36]	Ding et al. [21] Veugelers et al. [42]		Edmonds et al. [22]
**Various food group intake**		Davis & Carpenter [20]	An et al. [18] Laska et al. [31] Skidmore et al. [37] Jennings et al. [28]	Sturm & Datar [39]	Ho et al. [26]		
**Snack/junk food, candy intake**		Buck et al. [19]					
**Sugar-sweetened beverage intake**			Pabayo et al. [35]				
**Fast food intake**		Fraser et al. [23] Timperio et al. [41]		Khan et al. [29]		Longacre et al. [33]	
**Diet quality**		He et al. [24]	Lamichhane et al. [30]		Veugelers et al. [42]		
**Energy and/or macronutrient intake**					Veugelers et al. [42] Wang & Shi [43]	Leung et al. [32]	
**Healthy vs unhealthy diet scores**			Smith et al. [38]				
**Food purchasing behavior**		He et al. [25]					

Table 3 shows the 16 studies that employed some kind of GIS based measures of community FE to capture the geographic relationship between residents’ homes and/or school and an array of food store types: supermarkets, convenience stores, FF outlets, and others. The GIS-based measures that were used assessed only constructs related to the availability (presence, counts and density) and accessibility (distance to the nearest food outlet) dimensions of the community FE. Of the 16 studies, two studies [28,35] used GIS-based methods to look at food store availability within a certain buffer zone from home; one study examined FF accessibility within a buffer from home [23], four studies examined both the availability and accessibility dimensions of food outlets and FF from home [27,30,37,40]; one study looked at both availability and accessibility of grocery stores within census tracts [34]; five studies measured both the availability and accessibility dimensions of some kinds of food outlets such as grocery stores, convenience stores and FF restaurants from respondents’ home and school; and the last 3 studies used GIS methods to assess the density and distance of food outlets, for instance unhealthy food stores – convenience stores, FF restaurants to school [20,38] and density of all food outlets (using counts) within 1.5 km of school service area [19]. These studies used a wide range of buffer distances from home or school ranging from 0.1 mile [18] to 3000 m [31] to characterize the community FE. One study did not use a buffer distance from residents’ homes, but looked at grocery store availability within the census tract [34]. Another study focused on the food supply around schools calculated using the number of stores and restaurants divided by the number of residents per area [19].

**Table 3:** The associations between the food environment and dietary intake in studies using GIS-based measures to capture community food environment exposure

**Author (Year)**	**Sample size (n)**	**Specific exposure reported**	**FF outcome**	**Results**
Jago et al. [27]	204	Distance to food outlets from home and density of food outlets within a 1 mile radius of participant home	Fruit (17 types), 100% juice (4 types) and vegetable (17 types) consumption	- Distance to the nearest small food store was positively associated with fruit and juice consumption (z = 3.07, p = 0.002).
				- Distance to the nearest FF restaurant was negatively associated with fruit and juice consumption (z = −2.76, p = 0.006).
Mushi-Brunt et al. [66]	797	Grocery store density within census tract and distance to grocery store	Daily FV intake	There were no statistically significant associations between number of grocery stores and distance to grocery store and mean number of FV servings. However, children in low poverty NBHs (where more grocery stores were available and closer to one’s home) ate more FV per day than children in high poverty NBHs.
Timperio et al. [40]	5-6: 340 10–12: 461	Availability of five types of food outlets within 800 m of home and distance to the closet food outlet	Weekly fruit (14 types) and vegetable (13 types) consumption	- The more FF outlets (OR = 0.82, 95% CI 0.67-0.99) and convenience stores (OR = 0.84, 95% CI 0.73-0.98) close to home, the lower the likelihood of consuming fruit > = 2 times/day.
				- An inverse association between density of convenience stores and the likelihood of consuming vegetables > = 3 times/day (OR = 0.84, 95% CI 0.74-0.95).
				- The likelihood of consuming vegetables > = 3 times/day was greater the farther children lived from a supermarket (OR = 1.27, 95% CI 1.07-1.51) or FF outlet (OR = 1.19, 95% CI 1.06-1.35).
Fraser et al. [23]	4827	FF accessibility score using 1000 m buffer from home	FF consumption	Accessibility of FF outlets and consumption varied with space. In rural areas increased accessibility was associated with increased consumption, while in some urban areas increased accessibility was associated with lack of consumption (data not shown).
He et al. [25]	810	Junk food density within 1 km of home and school; distance from home and school to closest FF restaurant and convenience store	Food purchasing behavior (FF and convenience store)	- Close proximity (<1 km) to the nearest FF outlet (OR = 1.5, 95% CI 1.1-2.1), convenience store (OR = 2.5, 95% CI 1.5-3.6) in the home NBH increased the likelihood of purchasing from these food locations at least once per week by adolescents (p<0.05).
				- High density of FF outlets within 1 km buffer of the school (OR = 1.4, 95% CI 1.1-1.7) and home (OR = 1.6, 95% CI 1.1-2.3) associated with increased purchasing of FF by adolescents.
Pabayo et al. [35]	2,114	Food outlet availability within 1 km of home	Beverage intake (the number of servings for each beverage - soft drinks, fruit juice, milk and water - over an average day or over an average week)	- Living within 1 km of a grocery store, children were less likely to consume regular soft drinks (children who had 1–3 grocery stores RR = 0.84, 95% CI 0.73-0.96); children who had > = 4 grocery stores RR = 0.64, 95% CI 0.42-0.98).
Timperio et al. [41]	5-6: 343 10–12: 463	Distance from home to closest outlet, density and availability of FF/takeaway within 800 m of home and school	Weekly consumption of FF/takeaway	- Only density of stores close to home was associated with consuming takeaway/FF at least once weekly (OR = 0.97, 95% CI 0.95-1.00).
				- No associations between availability en route to school and likelihood of consuming takeaway/FF at least once weekly.
				- No association between distance to closet food outlet and consumption of FF/takeaway.
An et al. [18]	5-11: 8226	Food outlet counts and density at several distances (varying from 0.1, 0.5, 1.0 to 1.5 miles) from a respondent’s home and school	Daily consumption of FV, 100% juice, milk, soft drinks, high sugar foods and FF	- No robust relationship found between food environment and consumption (a few significant results were sensitive to small modeling changes and more likely to reflect chance than true relationships).
	12-17: 5236			
He et al. [24]	810	Junk food density within 1km of home and school; distance from home and school to closest FF restaurant and convenience store	Overall diet quality – Healthy Eating Index	- Healthy Eating Index (HEI) higher for those living further than 1km from the closest convenience store (p<0.01), and attending a school further than 1km from convenience (p<0.01) or FF locations (p<0.05).
				- Schools with 3 or more FF outlets within 1km had lower HEI scores than those with none in surrounding area (p<0.05).
Jennings et al. [28]	1,669	Food outlet (BMI-healthy and unhealthy) availability within 800 m of home	Food group intake (food and drink diary)	Unhealthy food intake (fizzy drinks 15.3%, p = 0.04 and noncarbonated fruit drinks 11.8%, p = 0.03) were associated with availability of BMI-unhealthy food outlets.
Lamichhane et al. [30]	359	Accessibility and availability of supermarkets (4 and 6 miles) and FF (1 mile) to home	The overall dietary intake quality composed of 8 food groups: grains, vegetables, fruit, dairy, meat, nut/seeds/legumes, fats/oils, sweets (DASH adherence score) and food groups	- The DASH adherence score significantly decreased by 0.29 for every mile increase in distance to the nearest supermarket (95% CI −0.57 - −0.02) and by 0.30 for every mile increase to 3 nearest supermarkets (95% CI − 0.59 - −0.008).
				- The DASH score significantly increased for each additional supermarket/square mile (estimate difference= 5.25, 95% CI 0.51-9.98).
				- None of the FF outlets accessibility/availability measures were significantly associated with the DASH score.
				- Intake of FV and low fat dairy significantly decreased as an individual resided at greater distance from the 3 nearest supermarkets (fruit: estimated difference: −0.06, 95% CI −0.12 - −0.003; vegetables: estimated difference: −0.03, 95% CI −0.08 - −0.01; low-fat dairy: estimated difference: −0.04, 95% CI −0.07 - −0.01).
				- Intake of low fat dairy increased, and meat and sweets decreased as an individual resided a greater distance from the 3 nearest FF outlets (low-fat dairy: estimated: 0.03, 95% CI 0.01-0.06; meat: estimated difference: −0.04, 95% CI −0.08- −0.01; sweets: estimated difference: −0.04, 95% CI −0.08 - −0.003).
Laska et al. [31]	349	Distance and density of all food outlets to home and school (800, 1600 and 3000 m buffer zones)	Food group intake	- SSB intake was negatively associated with distance from home to the nearest restaurant (beta=−0.007, 95% CI −0.01 - −0.003) or grocery store (beta=−0.005, 95% CI −0.01 - −0.001) with greater distance associated with less consumption.
				- SSB consumption was positively associated with food outlet density across a wide range of measures, including having at least one FF restaurant, restaurant of any kind, convenience store, grocery store or any retail facility within a 1600 m residential network buffer, and presence of a restaurant within 800 m. School level association: - There was no significant association (p>0.1) between energy, dietary fat, FV, vegetables alone or FF and convenience store purchasing and GIS variables.
Skidmore et al. [37]	2064	Distance to nearest food outlet, density/km^2^ of food outlets within 800 m buffer zone of a child’s home	Food choices (consisted of 15 common foods)	- Both distance and density of local food outlets were associated with food intake in children. - Living further away from a supermarket increased portions of fruit (0.11 portions/week/km increase, p<0.05) and vegetables (0.11 portion/week, p<0.05) consumed - Living closer to convenience stores was also associated with an increased consumption of potato chips, chocolate and white bread. - Density of supermarkets was associated with both an increase in vegetable intake (0.31 portions/week, p<0.05) and unhealthy foods.
Buck et al. [19]	384	Unhealthy food supply around schools calculated using number of stores and restaurants divided by number of residents per 1.5 km school service area	Junk food and simple sugar food consumption per week	Unhealthy food supply was not significantly clustered around schools.
Davis & Carpenter [20]	<50,000	Proximity and distance of FF to school, density of FF restaurants within a half-mile radius of the youth’s school	Soft drinks FV, juice, fried potato consumption in past 24 hrs	Students with FF restaurants near their school consumed fewer vegetables (beta= −0.02, 95% CI −0.03 - 0.00) or fruit (beta= −0.02, 95% CI −0.04 - 0.00) or juice (beta=−0.02, 95% CI −0.03 - 0.00) and more servings of soda (AOR= 1.05, 95% CI 1.00-1.11).
Smith et al. [38]	2001: 1382 2005: 524	Density of food outlets within 400 and 800 m network distance from school	Healthy and unhealthy diet scores calculated – daily value	- Positive relationships between distances travelled to grocers within 800m and healthy diet scores (0.003, 95% CI 0.001-0.006)
				- Significant negative relationship between proximity to takeaways and unhealthy diet scores.
				- No statistically significant relationship between count of food outlets and diet scores.

Table 3 also depicts the degree to which these different dimensions of access were found to be associated with dietary outcomes. The availability measures were the most common way to measure the community FE; they were used in 15 out of 16 studies, 11 of which showed a significant association between geographic availability and dietary outcomes, while the other 4 studies revealed only null associations [18,19,34,38]. Three of the eleven studies showed some associations but not always in a consistent direction [27,31,37]. For example Laska et al. [31] reported that SSB consumption was positively associated with food outlet density across a wide range of measures, including having at least one FF restaurant, a restaurant of any kind, a convenience store and grocery store or any retail facility within a 1600m residential network buffer, and the presence of a restaurant within 800m.

Measures representing food accessibility demonstrated some inconsistent relationships with dietary outcomes. Among the 14 of 16 studies that examined distance to a food store in relation to diet, three revealed null associations [18,34,41]. Five of the remaining eleven studies showed associations but not always in a consistent direction [23,27,37,38,40]. For instance, Timperio et. al. [40] found that the likelihood of consuming vegetables > = 3 times/day was greater not only the farther children lived from a supermarket (OR = 1.27, 95% CI 1.07-1.51) and but also the farther children lived from FF outlet (OR = 1.19, 95% CI 1.06-1.35). Skidmore et al. [37] reported not only that living further away from a supermarket increased portions of fruit (0.11 portions/week/km increase, p < 0.05) and vegetables (0.11 portion/week, p < 0.05) consumed, but also living closer to convenience stores was also associated with an increased consumption of potato chips, chocolate and white bread.

Table 4 examines the 4 studies that used participant reported measures to characterize the community FE. Studies that used measures of perceived food availability [26,43] were particularly consistent in showing a relationship with dietary outcomes (2/2 studies). For example, Ho et al. [26] reported that perceived availability of FF outlets and convenience stores was positively associated with moderate/high consumptions of FF (OR_ff_: 1.10; OR_con_ = 1.15) and junk food/soft drinks (OR_ff_ = 1.10; OR_con_ = 1.10). They also observed a significant negative association between the perceived availability of a restaurant with intakes of FV (OR_veg_ = 0.87 and OR_fruit_ = 0.83). Self-reported proximity of NBH food outlets was found to be positively associated with FV consumption only in one [42] out of two studies [21,42] where it was measured. For example, relative to NBHs with the poorest access to food outlets, children with the best access to food outlets consumed more FV (IR = 1.08, 95% CI 1.01-1.15) [42].

**Table 4:** The association between food environment and dietary intake in studies using self-reported measures to capture food environment exposure

**Author (Year)**	**Sample size (n)**	**Specific exposure reported**	**FF outcome**	**Results**
Ding et al. [21]	458	Self-report proximity of NBH food outlets (healthful food outlets vs. less healthful food outlets)	Daily FV intake	FV intake was not significantly associated with community food environment.
Veugelers et al. [42]	5200	Access to food stores from home (self –reported)	Diet quality index, daily servings of FV, energy from fat	Relative to NBHs with poorest access to shops, children with best access to shops consumed more FV (IR = 1.08, 95% CI 1.01-1.15), less dietary fat (IR = 0.51, 95% CI 0.33-0.78) and had a higher Diet Quality Index (DQI) (IR = 2.26, 95% CI 1.09-4.69).
Ho et al. [26]	24,796	Self-reported presence of food outlets (FF, convenience stores and Western and Hong Kong style restaurants) near home within 5 minute walking	Consumption of 4 food groups: FF, junk food/soft drink, fruit, and vegetables	- Perceived availability of FF outlets and convenience stores positively associated with moderate/high consumptions of FF (OR_ff_: 1.10; OR_con_ = 1.15) and junk food/soft drinks (OR_ff_ = 1.10; OR_con_ = 1.10).
				- Significant negative association between the perceived availability of restaurants with intakes of FV (OR_veg_ = 0.87 and OR_fruit_ = 0.83). - Positive relationship between reporting FF outlets with intake of junk food/soft drinks observed only in boys.
Wang & Shi [43]	2004: 373 2006: 303	Self-reported NBH density of food outlets within 5 km	Macronutrient and caloric intake	Density of wet markets positively associated with all four different measurements of nutrition intake.

In Table 5 we present the three studies that used an index of food prices for the area in which participants lived and the three studies that used a store audit measure – including the presence of specific food groups and shelf-space dedicated to these in food stores and restaurants – to assess the relationship between community and consumer food environment and diet. Higher prices of FF were associated with at least one measure of dietary health in two [29,36] out of the three studies in which they were measured. Two studies that used an index of FV prices were consistent in showing a negative relationship with FV consumption (2/2 studies) [36,39].

**Table 5:** The association between food environment and dietary intake in studies using other measures (food prices and store audits) to capture food environment exposure

**Author (Year)**	**Sample size (n)**	**Specific exposure reported**	**FF outcome**	**Results**
Powell et al. [36]	47,675	FV and FF prices from Cost of Living Index	Daily FV consumption	- A dollar increase in the price of FF is statistically significantly associated with a reduction in frequent consumption of FV by 6.7 percentage points (p<0.001).
				- A dollar increase in the price of FV is estimated to decrease FV consumption by 6.3 percentage points (z = 2.05).
Khan et al. [29]	11,700	FF prices from the Cost of Living Index and food outlet density	FF consumption in the past 7 days	- Higher FF prices were associated with lower childhood FF consumption (beta = −0.527, p<0.05). - FF restaurant outlet densities were significantly associated with FF consumption patterns (beta = 0.025, p<0.05).
Sturm & Datar [39]	4896	Price indices for meat, FV, dairy and FF calculated from Cost of Living Index	Consumption of FV, milk, soft drinks and FF in the past 7 days	- Lower real prices for FV predict higher intake frequency (a 1SD increase in the price index for FV is associated with a 0.82 times per week reduction in the frequency of consumption of FV), higher dairy prices predict lower milk consumption (a 1 SD increase in dairy prices predicts a reduction in milk consumption of two-thirds of a glass per week), and increased meat price predicts increased milk consumption.
				- The effects on FF and SSB are small and generally insignificant.
Edmonds et al. [22]	90	FV, 100% juice availability and shelf space in food stores and restaurants around home	FV (including fried potatoes), juice and consumption per day	Significant positive correlations were found between restaurant juice (*r* = 0.61, *p* < 0.05) and vegetable availability (*r* = 0.53, *p* = 0.10) and Boy Scouts’ reported consumption of juice and vegetables, - but no relationships were detected with grocery store availability.
Longacre et al. [33]	1,547	Availability of FF outlets (onsite audit)	FF intake in past week	Adolescent who lived in towns with > = 5 FF outlets were about 30% more likely to eat FF compared to those in town with no FF outlets (RR = 1.29, 95% CI 1.10 - 1.51).
Leung et al. [32]	215	“Food and retail” scale - Food outlet audit on random street segments within 0.25 mile of home	Total energy intake	Inverse relationship between prevalence of food and retail locations and total energy intake (for a one quartile increase, OR=0.84, 95% CI 0.74-0.96).

Results for measures of fruit, juice and vegetable availability and shelf space in food stores and restaurants around a child’s home also showed significant positive correlations with FV consumption in Boy Scouts. Specifically, significant positive correlations were found between restaurant juice (*r* = 0.61, *p* < 0.05) and vegetable availability (*r* = 0.53, *p* = 0.10) and Boy Scouts’ reported consumption of juice and vegetables [22]. Two studies [32,33] used store audit measures to assess the availability of FF outlets [33] and food outlets [32] with FF intake and total energy intake respectively. These showed some evidence of a relationship with FE features. For instance, adolescents who lived in towns with > = 5 FF outlets were about 30% more likely to eat FF compared to those in town with no FF outlets (RR = 1.29, 95% CI 1.10 - 1.51) [33].

The vast majority of the studies were cross-sectional in design, except for two articles reporting on longitudinal studies [29,38]. One longitudinal study from Khan et al. [29] used FF price to characterize the consumer FE and found that higher FF prices were associated with lower childhood FF consumption (beta = −0.527, p < 0.05), and that FF restaurant outlet densities were significantly associated with FF consumption patterns (beta = 0.025, p < 0.05). The other longitudinal study used GIS to measure density and distance of food outlets from school [38]. The study found positive relationships between distances travelled to grocers within 800m and healthy diet scores (0.003, 95% CI: 0.001, 0.006), and a significant negative relationship between proximity to takeaways and unhealthy diet scores.

## Discussion

To our knowledge, this review is the first to focus on the influence of the community and consumer nutrition environments on the diet of children. This systematic review of 26 studies of the relationship between the food environment and dietary intake in children found moderately strong evidence in support of the hypothesis that community and consumer nutrition environments may influence diet. Specifically, twenty-two out of twenty-six studies showed at least one positive association between the food environment exposure and diet outcome. Four studies reported only null associations with dietary outcomes. Studies that employed *GIS-based measures* were more common than those using other measures, however these studies less consistently reported a significant relationship between the food environment measure and dietary outcomes in the expected direction (i.e. unhealthy food environment characteristics were associated with some characteristics of poor dietary intake). This finding is consistent with the review by Caspi et. al [11]. Among studies that relied on GIS-based measures to characterize the food environment, *measures of accessibility* (often operationalized as distance to the nearest food outlets) were somewhat less consistent in finding significant expected associations with dietary outcomes compared to *measures of availability. Self reported measures of availability* were more consistently associated with multiple dietary outcomes, while *self reported measures of store accessibility*, revealed a statistically significant association with multiple dietary outcome in only 1 out of two studies, and the magnitude of the association was very small [42]. *Measures of fruit and vegetables and fast food prices* based on regional price indices were consistently related to multiple dietary outcomes in all three studies that used these measures. *Food store audit studies* showed an association between availability of food outlets and consumption of fruit and vegetables, fast food intake or total energy intake.

Despite the relatively large number of studies on this topic, there is significant variability in their measurement of the community and consumer nutrition environment, as well in dietary assessment, and as such there is little comparability between studies. For example, we found wide variation in buffer sizes used ranging from 160 to 3000 meters, although the majority used either Euclidean or road network buffers in the range of 500 to 1000 meters which is consistent with recommendations for distances typically travelled by foot [45]. Also, only 6 studies (those in Table 5) used either indices of food prices or store audits to capture food environment exposures. We agree with others that these types of measures of the consumer nutrition environment are most promising for capturing a more nuanced picture of neighbourhood food environment exposure [46], especially combined with measurement of the community nutrition environment. Again, only four studies (those in Table 4) used self-reported measures (so perceived food environment) to examine exposure. While in most research areas self-report is not a preferred data collection method to direct measures, it may be that perceptions of the food environment are quite important for determining consumption patterns, and therefore the limited number of studies that use participant perceptions could be a limitation within the literature. Finally, like other reviews of food environment measurement studies [11,14] we found inconsistencies in the evidence examining the impacts of food environment on diet and argue that the lack of standard measurements that are comparable across studies impedes our ability to clarify whether and how food environments effect diet.

### Recommendations

Caspi et al.’s [11] and Kirkpatrick et al.’s [14] recommendations are relevant to the current review. We agree with the previous systematic review by Caspi et al. [11] that refining the measures used to capture dimensions of food access is a priority for future research examining the food environment (or more specifically the community and consumer nutrition environments) – diet relationship. Kirkpatrick et al. [14] made recommendations focused on diet measurement in food environments research, and these are also applicable here. We make the following additional recommendations for future research:

1) We need to not only measure observable parameters of the food environment, but also capture the perceived food environment for children in order to better understand issues such as access, distances travelled, and aspects of the food environment that pose particular challenges to this group. There is some qualitative research available on the ‘foodscapes’ of children and their food purchasing decisions [16,17,47,48], and this literature in particular should be a starting point for future research in this area. Qualitative studies will also enable the further development of some of the ‘understudied measures’ described by Caspi et al. [11], such as food quality and food acceptability. There are a growing number of studies using qualitative methods to better understand perceptions of and interactions with the food environment in adults [49-52], but there continue to be major gaps to be filled, especially when it comes to research on children. In order to inform this research, food environments researchers should begin by examining the small but important body of literature on family feeding [53,54], given that it may inform a more nuanced understanding of food access. Qualitative studies that follow participants for long periods of time (up to a year or more), include multiple forms of data collection including interviews, observation, and other methods, are particularly needed. As DeVault [55] and Short [56] have argued, the daily tasks of ‘feeding the family’ are implicit, and often difficult to describe, and would benefit from the depth of understanding that can be developed over time and using various methods.

2) Recently, Burgoine et al. [57] found food environment measures of density and proximity to be highly correlated, and concluded that the heterogeneity found in GIS-based exposure metrics within the published literature may not be as problematic as previously argued [58]. Therefore, it might be particularly important to focus future research on combining GIS-based objective measurement of the community food environment with self-report measures of the community food environment, as well as measures of the consumer food environment. Caspi et al. [11] highlighted the importance of combining both community and consumer nutrition environment measures [7] in order to study not only the geographical aspects of the food environment, but also what is actually available in food outlets, the food’s quality, price, and promotion. There are few studies that use in-store measures of the consumer nutrition environment in the food environment literature as a whole and we were only able to find three studies that were focused on children as the population of interest. There is a need to conduct more studies that combine the community and consumer nutrition environments, ideally using valid and reliable tools that can be adapted to multiple settings such as the Nutrition Environment Measures Survey for Stores and Restaurants (NEMS-S/R) combined with shelf space measures of healthy versus less healthy food options [59,60]. NEMS-R already examines children’s menus so it is a readily available tool for this type of research. Shelf space measures of displays near checkouts, as well as unhealthy foods placed at children’s eye level in stores, would make a useful contribution.

3) Studies that focus on children’s diet as the outcome of interest are particularly needed. Kirkpatrick et al. [14] thoroughly discussed the need for using better validated dietary assessment tools in food environments and diet research. That recommendation is relevant here, but in addition, we also argue that ensuring the appropriateness of tools for dietary assessment in children is a factor to consider. Dietary assessment is both challenging and expensive to do well and almost always relies on self-report in non-institutional settings. Tools that have been validated in children should be used, and attempts should be made to use tools that are being widely used in various contexts such as the Youth/Adolescent Questionnaire from the Harvard School of Public Health [42,61].

4) Future research should be informed by literature on how children interact with their food environments. Studies should focus on different age groups of children, based upon their differing levels of independence and mobility. For example, younger children (under the age of 10) are generally limited by their parents’ food choices. Older children, between 10–15 years, on the other hand, have some independence and mobility and may be more limited by what is available in their home and school neighbourhoods as they travel on foot [62]. Once adolescents reach driving age, their food environments may change. Each of these age groups should be studied separately in order to understand how food environments may impact them differently. While there is some qualitative [16] and some quantitative research in this area [17,47,48], there is a need for more of both to better understand how children interact with their community and consumer nutrition environments.

### Limitations

The large number of references after de-duplication meant that we were not able within a reasonable time frame to have all references reviewed for exclusion by two team members. This means that there is a greater likelihood that articles were excluded that should not have been. However, the fact that all reviewers were instructed to be overly inclusive at that first screening phase means that risk is somewhat reduced. Another possible limitation to this research is publication bias, or that articles showing significant associations are more likely to be published than those that do not. If publication bias is a problem in the academic literature on food environments, it is possible that our conclusions are over-stated.

## Conclusions

This review of the published literature found some evidence of the relationship between the community and consumer nutrition environments and dietary intake in children up to 18 years of age. There is wide variation in measures used to characterize both the community and consumer food environments and diet, and future research should work to decrease this heterogeneity. Studies should measure both the community and consumer nutrition environments (perhaps using combinations of spatial and in-store food outlet audit measures). Diet should also be measured comprehensively using well-validated tools, rather than relying on short screeners. There continues to be significant need for better understanding of the relationship between food environment characteristics and diet in children, particularly by degree of child independence (so therefore by different age groups) in order to ensure that efforts to improve the diets of children are effective.

## Competing interests

The authors declare that they have no competing interest.

## Authors’ contributions

RES contributed to designing the review, participated in the title and later abstract and further review for inclusion, contributed to data extraction and drafted parts of the manuscript. HL participated in the title and later abstract and further review for inclusion, contributed to data extraction and drafted parts of the manuscript. AG contributed to designing the review and developed the search strategies, conducted the searches and drafted parts of the manuscript. NM contributed to designing the review and provided a critical analysis of the manuscript. All authors read and approved the final manuscript.

## Pre-publication history

The pre-publication history for this paper can be accessed here:

http://www.biomedcentral.com/1471-2458/14/522/prepub

## Supplementary Material

Additional file 1 MEDLINE Search Strategy (OvidSP Interface). Click here for file

Additional file 2 Comprehensive Search Strategies. Click here for file
