# Supplementary material for: The community and consumer food environment and children’s diet: a systematic review
Source: BMC Public Health. 2014 May 29;14:522. doi: 10.1186/1471-2458-14-522 (PMC4048041; doi:10.1186/1471-2458-14-522)
Supplement: Additional file 1 — MEDLINE Search Strategy (OvidSP Interface). [file 1471-2458-14-522-S1.pdf]

Additional File 1: MEDLINE Search Strategy (OvidSP Interface)

1. Environment/
2. "nutrition environment*".mp.
3. "supermarket*".mp.
4. "grocery store*".mp.
5. "convenience store*".mp.
6. "food outlet*".mp.
7. Food Industry/
8. food services/
9. restaurants/
10. Food supply/
11. exp Food packaging/
12. exp Fast Foods/
13. "food environment*".mp.
14. "food desert*".mp.
15. "community garden*".mp.
16. "farmers market*".mp.
17. "food accessibility".mp.
18. foodscape*.mp.
19. "portion size*".mp.
20. "food price*".mp.
21. "food option*".mp.
22. "food availability".mp.
23. "food marketing".mp.
24. "food advertis*".mp.
25. "neighborhood food*".mp.
26. "neighbourhood food*".mp.
27. "corner store*".mp.
28. "food retail*".mp.
29. exp Food Dispensers, Automatic/
30. Food/ec
31. 1 or 2 or 3 or 4 or 5 or 6 or 7 or 8 or 9 or 10 or 11 or 12 or 13 or 14 or 15 or 16 or 17 or 18 or 19 or 20 or 21 or 22 or 23 or 24 or 25 or 26 or 27 or 28 or 29 or 30
32. exp adolescent/
33. exp child/
34. exp infant/
35. exp adolescent behavior/
36. exp child behavior/
37. exp Parents/
38. teen*.mp.
39. youth.mp.
40. "young people".mp.
41. prepubescent.mp.
42. pubescent.mp.

43. 32 or 33 or 34 or 35 or 36 or 37 or 38 or 39 or 40 or 41 or 42
44. Social Environment/
45. 31 or 44
46. exp Child Nutritional Physiological Phenomena/
47. exp Diet/
48. Eating/
49. exp Child Nutrition Sciences/
50. exp Nutritional Status/
51. exp Nutritional Requirements/
52. exp Child Nutrition Sciences/
53. exp Nutritive Value/
54. Feeding Behavior/
55. exp food habits/
56. exp food preferences/
57. "food choice*".mp.
58. exp Dietary Carbohydrates/
59. Beverages/
60. exp carbonated beverages/
61. exp energy drinks/
62. Fruit/
63. exp Vegetables/
64. exp Dietary Fats/
65. exp Sodium, Dietary/
66. "Sugar-sweetened beverage*".mp.
67. exp Dietary Fiber/
68. exp Dietary Proteins/
69. exp Health Food/
70. exp Meat/
71. 46 or 47 or 48 or 49 or 50 or 51 or 52 or 53 or 54 or 55 or 56 or 57 or 58 or 59 or 60 or 61 or 62 or 63 or 64 or 65 or 66 or 67 or 68 or 69 or 70
72. 43 and 45 and 71
73. limit 72 to (english language and humans and yr="1995 -Current" and journal article)
