# Supplementary material for: The community and consumer food environment and children’s diet: a systematic review
Source: BMC Public Health. 2014 May 29;14:522. doi: 10.1186/1471-2458-14-522 (PMC4048041; doi:10.1186/1471-2458-14-522)
Supplement: Additional file 2 — Comprehensive Search Strategies. [file 1471-2458-14-522-S2.pdf]

Additional File 2: Comprehensive Search Strategies

MEDLINE Database Search

1. Environment/
2. "nutrition environment*".mp.
3. "supermarket*".mp.
4. "grocery store*".mp.
5. "convenience store*".mp.
6. "food outlet*".mp.
7. Food Industry/
8. food services/
9. restaurants/
10. Food supply/
11. exp Food packaging/
12. exp Fast Foods/
13. "food environment*".mp.
14. "food desert*".mp.
15. "community garden*".mp.
16. "farmers market*".mp.
17. "food accessibility".mp.
18. foodscape*.mp.
19. "portion size*".mp.
20. "food price*".mp.
21. "food option*".mp.
22. "food availability".mp.
23. "food marketing".mp.
24. "food advertis*".mp.
25. "neighborhood food*".mp.
26. "neighbourhood food*".mp.
27. "corner store*".mp.
28. "food retail*".mp.
29. exp Food Dispensers, Automatic/
30. Food/ec
31. 1 or 2 or 3 or 4 or 5 or 6 or 7 or 8 or 9 or 10 or 11 or 12 or 13 or 14 or 15 or 16 or 17 or 18 or 19 or 20 or 21 or 22 or 23 or 24 or 25 or 26 or 27 or 28 or 29 or 30
32. exp adolescent/
33. exp child/
34. exp infant/
35. exp adolescent behavior/
36. exp child behavior/
37. exp Parents/
38. teen*.mp.
39. youth.mp.
40. "young people".mp.
41. prepubescent.mp.

42. pubescent.mp.
43. 32 or 33 or 34 or 35 or 36 or 37 or 38 or 39 or 40 or 41 or 42
44. Social Environment/
45. 31 or 44
46. exp Child Nutritional Physiological Phenomena/
47. exp Diet/
48. Eating/
49. exp Child Nutrition Sciences/
50. exp Nutritional Status/
51. exp Nutritional Requirements/
52. exp Child Nutrition Sciences/
53. exp Nutritive Value/
54. Feeding Behavior/
55. exp food habits/
56. exp food preferences/
57. "food choice*".mp.
58. exp Dietary Carbohydrates/
59. Beverages/
60. exp carbonated beverages/
61. exp energy drinks/
62. Fruit/
63. exp Vegetables/
64. exp Dietary Fats/
65. exp Sodium, Dietary/
66. "Sugar-sweetened beverage*".mp.
67. exp Dietary Fiber/
68. exp Dietary Proteins/
69. exp Health Food/
70. exp Meat/
71. 46 or 47 or 48 or 49 or 50 or 51 or 52 or 53 or 54 or 55 or 56 or 57 or 58 or 59 or 60 or 61 or 62 or 63 or 64 or 65 or 66 or 67 or 68 or 69 or 70
72. 43 and 45 and 71
73. limit 72 to (english language and humans and yr="1995 -Current" and journal article)

CINAHL Database Search

S396 (MH "Food Security")
S397 (MH "Food Handling")
S398 (MH "Food Dispensers, Automatic")
S399 (MH "Food Packaging+")
S400 (MH "Portion Size")
S401 (MH "Food Services")
S402 (MH "Restaurants")
S403 (MH "Food Labeling")
S404 (MH "Food Supply")

S405 (MH "Food Industry")
S406 (MH "Food/SD/EC")
S407 "fast food*"
S408 "food outlet*"
S409 "supermarket*"
S410 (MH "Shopping")
S411 "convenience store*"
S412 "neighbourhood food*"
S413 "neighborhood food*"
S414 (MH "Marketing+")
S415 "foodscape*"
S416 "food desert*"
S417 "food environment*"
S418 "nutrition environment*"
S419 S396 OR S397 OR S398 OR S399 OR S400 OR S401 OR S402 OR S403 OR S404 OR S405 OR S406 OR S407 OR S408 OR S409 OR S410 OR S411 OR S412 OR S413 OR S414 OR S415 OR S416 OR S417 OR S418
S420 (MH "Child+")
S421 (MH "Adolescence+")
S422 (MH "Adolescent Behavior")
S423 (MH "Child Behavior+")
S424 (MH "Parent-Child Relations+")
S425 (MH "Parents+")
S426 S420 OR S421 OR S422 OR S423 OR S424 OR S425
S427 (MH "Adolescent Nutrition")
S428 (MH "Child Nutrition")
S429 (MH "Diet+")
S430 (MH "Nutritional Status")
S431 (MH "Eating Behavior+")
S432 (MH "Health Food+")
S433 (MH "Dietary Carbohydrates+")
S434 (MH "Dietary Fats+")
S435 (MH "Dietary Fiber")
S436 (MH "Dietary Proteins")
S437 (MH "Fruit+")
S438 (MH "Meat+")
S439 (MH "Vegetables+")
S440 (MH "Carbonated Beverages")
S441 (MH "Energy Drinks")
S442 (MH "Fruit Juices+")
S443 (MH "Sports Drinks")
S444 "Sugar sweetened beverage*"
S445 (MH "Sodium, Dietary+")
S446 (MH "Snack Foods")

S447 S427 OR S428 OR S429 OR S430 OR S431 OR S432 OR S433 OR S434 OR S435 OR S436 OR S437 OR S438 OR S439 OR S440 OR S441 OR S442 OR S443 OR S444 OR S445 OR S446
S448 S395 AND S419 AND S447
Limiters - Published Date: 19950101-20130431; Peer Reviewed; Human; Language: English

Embase Database Search

1. exp food insecurity/
2. exp food security/
3. food handling/
4. exp catering service/
5. exp food industry/
6. exp food packaging/
7. exp food availability/
8. exp advertizing/
9. exp marketing/
10. exp fast food/
11. social environment/
12. exp community/
13. exp neighborhood/
14. "convenience store*".mp.
15. "supermarket*".kw.
16. foodscape*.mp.
17. "food desert*".mp.
18. "food environment*".mp.
19. "nutrition environment*".mp.
20. 1 or 2 or 3 or 4 or 5 or 6 or 7 or 8 or 9 or 10 or 11 or 12 or 13 or 14 or 15 or 16 or 17 or 18 or 19
21. exp adolescence/
22. exp adolescent/
23. exp child behavior/
24. child/
25. preschool child/
26. infant/
27. exp parent/
28. exp parental behavior/
29. exp child parent relation/
30. 21 or 22 or 23 or 24 or 25 or 26 or 27 or 28 or 29
31. Eating/
32. Nutrition/
33. Child Nutrition/
34. diet/
35. exp Feeding behavior/
36. exp nutritional value/
37. exp portion size/

38. exp nutritional status/
39. food intake/
40. beverage/
41. carbonated beverage/
42. energy drink/
43. exp fruit juice/
44. sports drink/
45. exp fruit/
46. exp vegetable/
47. exp dietary intake/
48. exp sodium intake/
49. exp dietary fiber/
50. exp health food/
51. exp meat/
52. 31 or 32 or 33 or 34 or 35 or 36 or 37 or 38 or 39 or 40 or 41 or 42 or 43 or 44 or 45 or 46 or 47 or 48 or 49 or 50 or 51
53. 20 and 30 and 52
54. limit 53 to (human and english language and yr="1995 -Current" and article)

GEOBASE Database Search

((((((((({nutrition environment*}) WN KY) OR (({food environment*}) WN KY)) OR (({food desert*}) WN KY)) OR (({foodscape*}) WN KY)) OR ((supermarket*) WN KY)) AND (1973-2013 WN YR)) OR ((((((({grocery store*}) WN KY) OR (({convenience store*}) WN KY)) OR (({food outlet*}) WN KY)) OR (({Food Industry}) WN CV)) OR (({food services}) WN KY)) AND (1973-2013 WN YR)) OR ((((((restaurant*) WN KY) OR (({Food supply}) WN CV)) OR (({Food packaging}) WN KY)) OR (({Fast Food*}) WN KY)) OR (({food labelling}) WN KY)) AND (1973-2013 WN YR)) OR ((((((({food accessibility}) WN KY) OR (({portion size*}) WN KY)) OR (({food price*}) WN KY)) OR (({food option*}) WN KY)) OR (({food availability}) WN CV)) AND (1973-2013 WN YR)) OR ((((((({food marketing}) WN KY) OR (({food advertis*}) WN KY)) OR (({neighborhood food*}) WN KY)) OR (({neighbourhood food*}) WN KY)) OR (({corner store*}) WN KY)) AND (1973-2013 WN YR)) OR (((((({food retail*}) WN KY) OR (({vending machine*}) WN KY)) OR (({Food security}) WN CV)) OR (({Food Market}) WN CV)) AND (1973-2013 WN YR)))) and ((((((((($adolescence) WN CV)) OR (($child) WN CV)) OR (($infant) WN KY)) OR (({adolescent behavio*}) WN KY)) OR (({child behavio*}) WN KY)) AND (1973-2013 WN YR)) OR ((((((Parent*) WN KY) OR ((teen*) WN KY)) OR (($youth) WN KY)) OR (({young population}) WN CV)) AND (1973-2013 WN YR)))) and (((((((((($diet) WN CV)) OR (($nutrition) WN CV)) OR (($eating) WN KY)) OR (({Nutritive Value}) WN CV)) OR (({Feeding Behavior}) WN CV)) AND (1973-2013 WN YR)) OR ((((((({food habit*}) WN KY) OR (({food preference*}) WN CV)) OR (({food choice*}) WN KY)) OR ((Beverage*) WN KY)) OR (({carbonated beverage*}) WN KY)) AND (1973-2013 WN YR)) OR (((((({Sugar-sweetened beverage*}) WN KY) OR (({Nutritional

Status}) WN CV)) OR (({nutritional requirement}) WN CV)) OR (({Food consumption}) WN CV)) AND (1973-2013 WN YR))))) +({ja} WN DT) AND ({english} WN LA)

ProQuest Public Health Database Search

((mesh(adolescent) OR mesh(child) OR mesh(infant) OR mesh(adolescent behavior) OR mesh(child behavior) OR mesh(Parents) OR all(teen*) OR all(youth*) OR all("young people") OR all(prepubescent) OR all(pubescent)) AND (mesh(Child Nutritional Physiological Phenomena) OR mesh(Diet) OR mesh(Eating) OR mesh(Child Nutrition Sciences) OR mesh(Nutritional Status) OR mesh(Nutritional Requirements) OR mesh(Nutritive Value) OR mesh(Feeding Behavior) OR mesh(food habits) OR mesh(food preferences) OR all("food choice*") OR mesh(Dietary Carbohydrates) OR mesh(Beverages) OR mesh(carbonated beverages) OR mesh(energy drinks) OR mesh(Fruit) OR mesh(Vegetables) OR mesh(Dietary Fats) OR mesh(Sodium, Dietary) OR all("Sugar-sweetened beverage*") OR mesh(Dietary Fiber) OR mesh(Dietary Proteins) OR mesh(Health Food) OR mesh(Meat)) AND (mesh(Environment) OR mesh(Social Environment) OR all("nutrition environment*") OR all("supermarket*") OR all("grocery store*") OR all("convenience store*") OR all("food outlet*") OR mesh(Food Industry) OR mesh(food services) OR mesh(restaurants) OR mesh(Food supply) OR mesh(Food packaging) OR mesh(Fast Foods) OR all("food environment*") OR all("food desert*") OR all("community garden*") OR all("farmers market*") OR all("food accessibility") OR all(foodscape*) OR all("portion size*") OR all("food price*") OR all("food option*") OR all("food availability") OR all("food marketing") OR all("food advertis*") OR all("neighborhood food*") OR all("neighbourhood food*") OR all("corner store*") OR all("food retail*") OR mesh(Food Dispensers, Automatic))) AND (at.exact("Article") AND stype.exact("Scholarly Journals") AND la.exact("ENG") AND pd(19950101-20130403)) AND PEER(yes)

PsycINFO Database Search

1. exp Environment/
2. "nutrition environment*".mp.
3. "food environment*".mp.
4. "food desert*".mp.
5. foodscape*.mp.
6. supermarket*.mp.
7. "grocery store*".mp.
8. "convenience store*".mp.
9. "food outlet*".mp.
10. "food service*".mp.
11. restaurant*.mp.
12. "food supply".mp.
13. "food packaging".mp.

14. "food labelling".mp.
15. "food labeling".mp.
16. "fast food*".mp.
17. "community garden*".mp.
18. "farmers market*".mp.
19. "food accessibility".mp.
20. "portion size*".mp.
21. "food price*".mp.
22. "food option*".mp.
23. "food availability".mp.
24. "food retail*".mp.
25. "corner store*".mp.
26. "neighbourhood food".mp.
27. "neighborhood food".mp.
28. "vending machine*".mp.
29. "food security".mp.
30. "food insecurity".mp.
31. "food marketing".mp.
32. "food advertising".mp.
33. exp neighborhoods/
34. 1 or 2 or 3 or 4 or 5 or 6 or 7 or 8 or 9 or 10 or 11 or 12 or 13 or 14 or 15 or 16 or 17 or 18 or 19 or 20 or 21 or 22 or 23 or 24 or 25 or 26 or 27 or 28 or 29 or 30 or 31 or 32 or 33
35. exp food/
36. exp food intake/
37. exp eating behavior/
38. exp diets/
39. exp nutrition/
40. "food habit*".mp.
41. exp food preferences/
42. exp "beverages (nonalcoholic)"/
43. "fruit juice*".mp.
44. "energy drink*".mp.
45. fruit*.mp.
46. "sugar-sweetened beverage*".mp.
47. exp carbohydrates/
48. vegetable*.mp.
49. exp lipids/
50. exp Sodium/
51. fiber.mp.
52. fibre.mp.
53. 35 or 36 or 37 or 38 or 39 or 40 or 41 or 42 or 43 or 44 or 45 or 46 or 47 or 48 or 49 or 50 or 51 or 52
54. 34 and 53
55. limit 54 to (100 childhood or 120 neonatal or 140 infancy or 160 preschool age or 180 school age or 200 adolescence)
56. child*.mp.

57. youth.mp.
58. adolescen*.mp.
59. teen*.mp.
60. "young people".mp.
61. 56 or 57 or 58 or 59 or 60
62. 54 and 61
63. 55 not 62
64. 62 not 55
65. 55 or 62
66. limit 65 to (human and english language and "0110 peer-reviewed journal" and yr="1995 - Current")
67. limit 66 to "0400 empirical study"

Scopus Database Search

(((TITLE-ABS-KEY("nutrition environment*") OR TITLE-ABS-KEY("food environment*") OR TITLE-ABS-KEY("food desert*") OR TITLE-ABS-KEY("foodscape*") OR TITLE-ABS-KEY(supermarket*))) OR ((TITLE-ABS-KEY("grocery store*") OR TITLE-ABS-KEY("convenience store*") OR TITLE-ABS-KEY("food outlet*") OR TITLE-ABS-KEY("Food Industry") OR TITLE-ABS-KEY("food services"))) OR ((TITLE-ABS-KEY("food accessibility") OR TITLE-ABS-KEY("portion size*") OR TITLE-ABS-KEY("food price*") OR TITLE-ABS-KEY("food option*") OR TITLE-ABS-KEY("food availability"))) OR ((TITLE-ABS-KEY("food marketing") OR TITLE-ABS-KEY("food advertis*") OR TITLE-ABS-KEY("neighborhood food*") OR TITLE-ABS-KEY("neighbourhood food*") OR TITLE-ABS-KEY("corner store*"))) OR ((TITLE-ABS-KEY("food retail*") OR TITLE-ABS-KEY("vending machine*") OR TITLE-ABS-KEY("Food security") OR TITLE-ABS-KEY("food insecurity"))) OR ((TITLE-ABS-KEY(restaurant*) OR TITLE-ABS-KEY("Food supply") OR TITLE-ABS-KEY("Food packaging") OR TITLE-ABS-KEY("food labelling") OR TITLE-ABS-KEY("Fast Food*")))) AND (((TITLE-ABS-KEY(adolescen*) OR TITLE-ABS-KEY(child*) OR TITLE-ABS-KEY(infant*) OR TITLE-ABS-KEY("adolescent behavio*") OR TITLE-ABS-KEY("child behavio*"))) OR ((TITLE-ABS-KEY(parent*) OR TITLE-ABS-KEY(teen*) OR TITLE-ABS-KEY(youth) OR TITLE-ABS-KEY("young people")))) AND (((TITLE-ABS-KEY(diet) OR TITLE-ABS-KEY(nutrition) OR TITLE-ABS-KEY(eating) OR TITLE-ABS-KEY("Nutritive Value") OR TITLE-ABS-KEY("Feeding Behavio*"))) OR ((TITLE-ABS-KEY("food habit*") OR TITLE-ABS-KEY("food preference*") OR TITLE-ABS-KEY("food choice*") OR TITLE-ABS-KEY(beverage*) OR TITLE-ABS-KEY("carbonated beverage*"))) OR ((TITLE-ABS-KEY("Sugar-sweetened beverage*") OR TITLE-ABS-KEY("Nutritional Status") OR TITLE-ABS-KEY("Nutritional Requirement*")))) AND (LIMIT-TO(PUBYEAR, 2013) OR LIMIT-TO(PUBYEAR, 2012) OR LIMIT-TO(PUBYEAR, 2011) OR LIMIT-TO(PUBYEAR, 2010) OR LIMIT-TO(PUBYEAR, 2009) OR LIMIT-TO(PUBYEAR, 2008) OR LIMIT-TO(PUBYEAR, 2007) OR LIMIT-TO(PUBYEAR, 2006) OR LIMIT-TO(PUBYEAR, 2005) OR LIMIT-TO(PUBYEAR, 2004) OR LIMIT-TO(PUBYEAR, 2003) OR LIMIT-

TO(PUBYEAR, 2002) OR LIMIT-TO(PUBYEAR, 2001) OR LIMIT-TO(PUBYEAR, 2000) OR LIMIT-TO(PUBYEAR, 1999) OR LIMIT-TO(PUBYEAR, 1998) OR LIMIT-TO(PUBYEAR, 1997) OR LIMIT-TO(PUBYEAR, 1996) OR LIMIT-TO(PUBYEAR, 1995)) AND (LIMIT-TO(DOCTYPE, "ar")) AND (LIMIT-TO(LANGUAGE, "English")) AND (LIMIT-TO(SRCTYPE, "j"))

Sociological Abstracts Database Search

(Environment OR "nutrition Environment" OR "food environment*" OR "food desert*" OR "foodscape*" OR "supermarket*" OR "grocery store*" OR "convenience store*" OR "food outlet*" OR "Food Industry" OR "food services" OR "restaurant*" OR "Food supply" OR "Food packaging" OR "food labelling" OR "Fast Food*" OR "community garden*" OR "farmers market*" OR "food accessibility" OR "portion size*" OR "food price*" OR "food option*" OR "food availability" OR "food marketing" OR "food advertis*" OR "neighborhood food*" OR "neighbourhood food*" OR "corner store*" OR "food retail*" OR "vending machine*" OR "Food security" OR "food insecurity") AND (Adolescen* OR child* OR infant* OR "adolescent behavio*" OR "child behavio*" OR Parent* OR teen* OR youth OR "young people*" OR prepubescent OR pubescent) AND (Diet* OR Eating OR "Nutritive Value" OR "Feeding Behavio*" OR "food habits" OR "food preference*" OR "food choice*" OR "Dietary Carbohydrate*" OR "Beverage*" OR "carbonated beverage*" OR "energy drink*" OR Fruit* OR Vegetable* OR "Dietary Fat*" OR "Sodium" OR "Sugar-sweetened beverage*" OR "Dietary Fiber" OR "Dietary Protein*" OR "Health Food*" OR Meat* OR "Nutritional Status" OR "Nutritional Requirement*") AND (rtype.exact("Journal Article") AND pd(19950101-20130403) AND PEER(yes))

Web of Science Database Search*

#1 Topic=("nutrition environment*") OR Topic=("food environment*") OR Topic=("food desert*") OR Topic=("foodscape*") OR Topic=(supermarket*) *DocType=All document types; Language=All languages;*

#2 Topic=("grocery store*") OR Topic=("convenience store*") OR Topic=("food outlet*") OR Topic=("Food Industry") OR Topic=("food services")
DocType=All document types; Language=All languages;

#3 Topic=(restaurant*) OR Topic=("food supply") OR Topic=("food packaging") OR Topic=("food label*") OR Topic=("fast food*")
DocType=All document types; Language=All languages

#4 Topic=("community garden*") OR Topic=("farmers market*") OR Topic=("food accessibility") OR Topic=("portion size*") OR Topic=("food price*")
DocType=All document types; Language=All languages;

#5 Topic=("food option*") OR Topic=("food availability*") OR Topic=("food marketing") OR Topic=("food advertis*") OR Topic=("neighborhood food*")
DocType=All document types; Language=All languages;

#6 Topic=("corner store*") OR Topic=("food retail*") OR Topic=("vending machine*") OR Topic=("food security") OR Topic=("neighbourhood food*") OR Topic=("food insecurity")
DocType=All document types; Language=All languages;

#7 #6 OR #5 OR #4 OR #3 OR #2 OR #1
DocType=All document types; Language=All languages;

#8 Topic=(Adolescen*) OR Topic=(child*) OR Topic=(infant*) OR Topic=("adolescent behavio*") OR Topic=("child behavio*") OR Topic=(parent*) OR Topic=(teen*)
DocType=All document types; Language=All languages;

#9 Topic=(youth) OR Topic=("young people*") OR Topic=(prepubescent) OR Topic=(pubescent)
DocType=All document types; Language=All languages;

#10 #9 OR #8
DocType=All document types; Language=All languages;

#11 Topic=(Diet) OR Topic=(eating) OR Topic=("nutritive value*") OR Topic=("Feeding Behavio*") OR Topic=("food habit*") OR Topic=("food preference*") OR Topic=("food choice*")
DocType=All document types; Language=All languages;

#12 Topic=("Dietary Carbohydrate*") OR Topic=(beverage*) OR Topic=("carbonated beverage*") OR Topic=("energy drink*") OR Topic=(fruit*) OR Topic=(vegetable*) OR Topic=("dietary fat*")
DocType=All document types; Language=All languages;

#13 Topic=("Dietary sodium") OR Topic=("Sugar-sweetened beverage*") OR Topic=("Dietary Fiber") OR Topic=("Dietary Protein*") OR Topic=("Health Food*") OR Topic=(Meat*) OR Topic=("Nutritional Status") OR Topic=("Nutritional Requirements")
DocType=All document types; Language=All languages;

#14 #13 OR #12 OR #11
DocType=All document types; Language=All languages;

#15 #14 AND #10 AND #7
DocType=All document types; Language=All languages;

#16 #14 AND #10 AND #7
Refined by: Publication Years=(2012 OR 2007 OR 2013 OR 1999 OR 2011 OR 2006 OR 2001 OR 1995 OR 2010 OR 2005 OR 2003 OR 1996 OR 2009 OR 2004 OR 2000 OR 1997 OR 2008 OR 2002 OR 1998)
DocType=All document types; Language=All languages;

#17 #14 AND #10 AND #7
Refined by: Publication Years=(2012 OR 2007 OR 2013 OR 1999 OR 2011 OR 2006 OR 2001 OR 1995 OR 2010 OR 2005 OR 2003 OR 1996 OR 2009 OR 2004 OR 2000 OR 1997 OR 2008 OR 2002 OR 1998) AND Document Types=(ARTICLE)
DocType=All document types; Language=All languages;

#18 #14 AND #10 AND #7
Refined by: Publication Years=(2012 OR 2007 OR 2013 OR 1999 OR 2011 OR 2006 OR 2001 OR 1995 OR 2010 OR 2005 OR 2003 OR 1996 OR 2009 OR 2004 OR 2000 OR 1997 OR 2008 OR 2002 OR 1998) AND Document Types=(ARTICLE) AND Languages=(ENGLISH)
DocType=All document types; Language=All languages;

* The Search History only allowed 40 saved searches; solution was to do a series of mini-searches per search line.
